# Mebendazole Inhibits *Histoplasma capsulatum* In Vitro Growth and Decreases Mitochondrion and Cytoskeleton Protein Levels

**DOI:** 10.3390/jof9030385

**Published:** 2023-03-21

**Authors:** Marcos Abreu Almeida, Andrea Reis Bernardes-Engemann, Rowena Alves Coelho, Camila Jantoro Guzman Lugones, Iara Bastos de Andrade, Dario Corrêa-Junior, Simone Santiago Carvalho de Oliveira, André Luis Souza dos Santos, Susana Frases, Márcio Lourenço Rodrigues, Richard Hemmi Valente, Rosely Maria Zancopé-Oliveira, Rodrigo Almeida-Paes

**Affiliations:** 1 Laboratório de Micologia, Instituto Nacional de Infectologia Evandro Chagas, Fundação Oswaldo Cruz, Rio de Janeiro 21040-900, Brazil; 2 Laboratório de Biofísica de Fungos, Instituto de Biofísica Carlos Chagas Filho, Universidade Federal do Rio de Janeiro, Rio de Janeiro 21941-902, Brazil; 3 Laboratório de Estudos Avançados de Microrganismos Emergentes e Resistentes, Departamento de Microbiologia Geral, Instituto de Microbiologia Paulo de Goés, Universidade Federal do Rio de Janeiro, Rio de Janeiro 21941-902, Brazil; 4 Rede Micologia RJ, Fundação de Amparo à Pesquisa do Estado do Rio de Janeiro (FAPERJ), Rio de Janeiro 21941-901, Brazil; 5 Instituto Carlos Chagas, Fundação Oswaldo Cruz, Curitiba 81350-010, Brazil; 6 Laboratório de Toxinologia, Instituto Oswaldo Cruz, Fundação Oswaldo Cruz, Rio de Janeiro 21040-900, Brazil

**Keywords:** antifungal activity, benzimidazole, drug repurposing, histoplasmosis, proteomics

## Abstract

Histoplasmosis is a frequent mycosis in people living with HIV/AIDS and other immunocompromised hosts. Histoplasmosis has high rates of mortality in these patients if treatment is unsuccessful. Itraconazole and amphotericin B are used to treat histoplasmosis; however, both antifungals have potentially severe pharmacokinetic drug interactions and toxicity. The present study determined the minimal inhibitory and fungicidal concentrations of mebendazole, a drug present in the NIH Clinical Collection, to establish whether it has fungicidal or fungistatic activity against *Histoplasma capsulatum*. Protein extracts from *H. capsulatum* yeasts, treated or not with mebendazole, were analyzed by proteomics to understand the metabolic changes driven by this benzimidazole. Mebendazole inhibited the growth of 10 *H. capsulatum* strains, presenting minimal inhibitory concentrations ranging from 5.0 to 0.08 µM. Proteomics revealed 30 and 18 proteins exclusively detected in untreated and mebendazole-treated *H. capsulatum* yeast cells, respectively. Proteins related to the tricarboxylic acid cycle, cytoskeleton, and ribosomes were highly abundant in untreated cells. Proteins related to the nitrogen, sulfur, and pyrimidine metabolisms were enriched in mebendazole-treated cells. Furthermore, mebendazole was able to inhibit the oxidative metabolism, disrupt the cytoskeleton, and decrease ribosomal proteins in *H. capsulatum*. These results suggest mebendazole as a drug to be repurposed for histoplasmosis treatment.

## 1. Introduction

Histoplasmosis is a serious fungal infection caused by the inhalation of microconidia from the dimorphic fungus *Histoplasma capsulatum*. Histoplasmosis can range from an asymptomatic infection to a life-threatening disseminated disease. In more serious cases, usually in individuals who are immunocompromised such as people living with HIV/AIDS (PLWHA) or those who take immunosuppressant medications, histoplasmosis can disseminate, leading to considerable morbidity and mortality [1]. Delay in diagnosis, limited access to antifungals, and drug resistance are directly associated with high mortality from HIV-associated histoplasmosis, especially in low- and middle-income countries [2].

Published treatment recommendations are specific to the different clinical forms of histoplasmosis; however, the antifungal drugs used are generally itraconazole and the different formulations of amphotericin B [3,4]. The standard antifungal regimen for disseminated histoplasmosis is induction therapy with amphotericin B, followed by consolidation therapy with itraconazole [5].

Nephrotoxicity is one important side effect of amphotericin B. About 80% of the patients will develop toxicity problems or kidney damage during the infusion of the drug [6]. In addition, amphotericin B is administered intravenously in a hospital setting, which requires minimal infrastructure [7]. In Europe, a 15-day intravenous treatment with liposomal amphotericin B is estimated to cost from €10,000 to €20,000 [8]. In Brazil, the daily average cost of treatment with liposomal amphotericin B was US$ 715.35 in an inpatient regimen [9]. The drug–drug interactions and the side effects are major issues associated with itraconazole therapy. Itraconazole presents drug interactions with agents used in HIV-antiretroviral therapy, such as nevirapine [10], and with rifampicin, used for tuberculosis treatment [11]. Common side effects of itraconazole include gastrointestinal disturbances, hepatic function disorders, nausea, mild diarrhea, vomiting, and abdominal pain [12].

Alternatively, according to the Clinical Practice Guidelines for the Management of Patients with Histoplasmosis, updated in 2007 by the Infectious Diseases Society of America, other azoles (fluconazole, voriconazole, ketoconazole, and posaconazole) are second-line alternatives to itraconazole in the treatment of histoplasmosis [13]. However, treatment failure may confer resistance, as described for fluconazole [14] and voriconazole [3]. The use of ketoconazole is unrecommended, since there is no action on the disseminated form of the disease [15], in addition to its toxicity. Although posaconazole demonstrated promising activity in in vitro and in vivo trials, cases of hypokalemia and metabolic alkalosis may occur in patients with high serum posaconazole levels [16,17]. Therefore, the search for new drugs for histoplasmosis treatment is urgent.

Drug repurposing brings a much faster response than developing a new drug, in addition to saving costs [18]. Studies involving drug repurposing to improve the therapeutic arsenal for histoplasmosis point to sorafenib, an antineoplastic agent indicated for the treatment of renal carcinoma, as a promising candidate. This drug inhibited *Histoplasma* growth in vitro and in a macrophage infection model with low host cell cytotoxicity [19]. Another study points to nitrofuran and indole derivatives as promising drugs for histoplasmosis treatment [20]. Nitrofurans are described as antibiotics with a broad spectrum of action [21] and chemical compounds containing indole rings have been explored in different applications, such as cancer, microbial and viral infections, as well as inflammation [22,23].

To improve the knowledge of possible drugs with repurposing potential for histoplasmosis treatment, we performed a screening of the National Institutes of Health (NIH) Clinical Collection, in order to search for possible drugs with anti-*H. capsulatum* activity. Furthermore, proteomics was used to investigate metabolic changes driven by a selected drug with potential use for other mycoses that can be misdiagnosed or occur concomitantly with histoplasmosis.

## 2. Materials and Methods

### 2.1. Fungal Strains and Growth Conditions

*H. capsulatum* G217B (ATCC 26032) reference strain was used throughout the study. In addition, other 10 *H. capsulatum* strains, of clinical or environmental origin, were included in antifungal susceptibility experiments. All isolates were identified by macroscopic morphology of colonies and microscopic examination, followed by partial DNA sequencing of selected genes [24]. All isolates are maintained at the INI/Fiocruz fungal collection. Fungal strains were kept in the mycelial form on Potato Dextrose Agar (PDA) (Becton, Dickinson and Company, Sparks, MD, USA) at 25 °C, and the dimorphism was demonstrated by conversion to the yeast-like form on ML-Gema agar medium [25] at 37 °C.

### 2.2. Screening of the NIH Clinical Collection 

The NIH Clinical Collection (NCC) consists of a repository of 727 small molecules, which have been in phase I-III clinical trials, diluted in dimethyl sulfoxide (DMSO) and distributed at 10 mM in 96-well plates. The screening was performed as described for *Cryptococcus neoformans* [26], but using the reference G217B *H. capsulatum* strain. Growth was assessed by the absorbance at 540 nm after 72 h of fungal growth at 37 °C using the SpectraMax Plus spectrophotometer (Molecular Devices, San Jose, CA, USA). The NCC screening was performed three times on different days. Small molecules that consistently reduced fungal survival by at least 50% as compared to the drug-free control wells with 0.5% *v*/*v* DMSO and presented a possible therapeutic usefulness for histoplasmosis were selected for minimal inhibitory concentration (MIC) determinations. These molecules, hereinafter, will be referred to as hit drugs. The percent survival was determined according to the following formula: percent survival = ((OD1 − OD2)/(OD3 − OD2)) × 100, where OD1 = fungal optical density in the presence of the drug; OD2 = optical density of the control well without fungal inoculum; and OD3 = optical density of the fungal growth control well without any drug.

### 2.3. Minimal Inhibitory Concentration (MIC) Determinations

The MIC of hit drugs against the *H. capsulatum* strains was determined by the broth microdilution method according to the EUCAST guideline [27], with few modifications to test this dimorphic fungus. Two-fold serial dilutions of stock solutions of hit drugs (4 mM) in DMSO were performed to obtain the final testing concentrations, ranging from 20 to 0.04 µM. Inocula of 1 × 10^6^ yeast-like cells/mL were prepared in sterile saline after incubation on ML-Gema for five days at 37 °C. Fungal suspensions were then added to the wells containing the different concentrations of the drug at a 1:1 proportion. A drug-free control well with 0.5% *v*/*v* DMSO (positive control) and a cell-free control well (negative control) were included for each strain. Plates with 5 × 10^5^ yeast-like cells/well were incubated at 35 °C for five days. The MIC was visually determined as the lowest drug concentration that completely inhibited fungal growth compared to the drug-free control. MIC determinations were performed in three independent experiments. At this point, drugs without antifungal action against all *H. capsulatum* strains tested were excluded from the analyses. 

### 2.4. Fungicidal Activity

Five microliters from each well without visual fungal growth in the MIC determination assay (described above) were plated on PDA to assess whether the antifungal activity of the drug was fungicidal or fungistatic. This led to a detection limit of 200 cfu/mL, which equals 2.3 log_10_ cfu/mL. After incubation for 21 days at 25 °C, colony growth was verified, and the macro- and micromorphological characteristics were analyzed [28] to check if the colonies were compatible with *H. capsulatum*. The minimal fungicidal concentration (MFC) was determined, in three independent experiments, as the lowest drug concentration without *H. capsulatum* growth on PDA after incubation. When the MFC/MIC ratio was 1 or 2, the drug was considered fungicidal. When higher than 2, the drug was classified as fungistatic [29].

### 2.5. Preparation of Protein Extracts

*H. capsulatum* G217B yeast-like cells, treated or not with mebendazole at a sub-inhibitory concentration (i.e., ½ × MIC value), were cultivated, in five biological replicates, in Ham’s F12 medium (Gibco, Grand Island, NY, USA) supplemented with cystine (8.4 mg/L), HEPES (6 g/L), glutamic acid (1 g/L), and glucose (18.2 g/L), pH 7.5. Untreated cells (controls) were given 0.5% *v*/*v* DMSO, since mebendazole was dissolved in this compound, at this final concentration. The initial inoculum was 1 × 10^7^ cells/mL, and cultures were maintained at 36 °C for 72 h in a rotatory shaker incubator (Tecnal, São Paulo, Brazil) at 150 rpm. Next, protein extracts were obtained as previously described [30]. Protein concentration was determined by the Bradford method [31] using bovine serum albumin (Sigma-Aldrich, Steinheim, Germany) for standard calibration curves, and the Bio-Rad Protein Assay Dye Reagent Concentrate (Bio-Rad, Hercules, CA, USA) for the colorimetric reaction. The overall protein heterogeneity was evaluated by 12% SDS-PAGE [32]. Subsequently, samples were dried on a centrifugal vacuum concentrator (ThermoFisher, Waltham, MA, USA) and stored at −20 °C.

### 2.6. Sample Processing for Shotgun Proteomics

For each sample, a volume containing 100 μg of protein was dried on the centrifugal vacuum concentrator. Next, samples were suspended in 20 μL of 0.4 M ammonium bicarbonate and 8 M urea, followed by the addition of 5 μL of 0.1 M dithiothreitol and incubation at 37 °C for 30 min. Then, 5 μL of 0.4 M iodoacetamide were added and incubated for 15 min at room temperature in the dark. Samples were diluted by the addition of 130 μL of Milli-Q water followed by trypsin (Promega, Madison, WI, USA) addition at 1/50 (*m*/*m*) of enzyme to substrate and sequential incubation for 16 h at 37 °C and 45 min at 56 °C. The reaction was stopped with 20 μL of 10% (*v*/*v*) formic acid (FA). Samples were desalted with in-lab generated columns packed with Poros R2 resin (Life Technologies, Carlsbad, CA, USA). Columns were initially activated with 2× 20 µL 100% acetonitrile (CH_3_CN), followed by equilibration with 4× 20 µL 1% (*v*/*v*) trifluoroacetic acid (TFA). Samples were applied to the columns, followed by washing with 5 × 20 µL of 0.1% TFA solution. Finally, each sample digest was eluted (4× 20 µL of 0.1% TFA in 70% CH_3_CN) to a new vial. Samples (80 µL per tube) were dried and stored at −20 °C until use. For mass spectrometry analysis, each sample was suspended in 20 μL of 1% FA, and its peptide concentration was estimated by absorbance measurement at 280 nm (1.0 absorbance unit ≅ 1.0 μg/μL) on a NanoDrop 2000 spectrophotometer (ThermoFisher). All samples were normalized to 0.5 μg/μL for the next analysis.

### 2.7. Mass Spectrometry Analysis

The desalted tryptic digests from five biological replicates from either mebendazole-treated or -untreated *H. capsulatum* G217B strain were analyzed in two technical replicates by reversed-phase nano-chromatography coupled to high-resolution nano-electrospray ionization mass spectrometry. Chromatography was performed using an Easy-nLC 1200 system (Thermo Fischer Scientific, Waltham, MA, USA). Samples (2 µL per run) were initially applied, at 2 μL/min of 0.1% (*v*/*v*) FA in water, to a 2-cm-long Thermo Acclaim trap column (75 µm inner diameter; 3 µm beads). Next, peptides were submitted to chromatographic separation on a 38-cm-long column (75 μm i.d.), packed with ReproSil-Pur C18-AQ 120 Å 1.9 μm matrix (Dr. Maisch GmbH, Germany) directly onto a self-pack 10 um PicroFrit empty column (New Objective, Littleton, MA, USA). Fractionation was performed at 200 nL/min with 0.1% (*v*/*v*) FA in water and 0.1% (*v*/*v*) FA in 80% CH_3_CN in water as mobile phases A and B, respectively. Elution was carried out from 2% to 30% B in 120 min; up to 45% B in 40 min; up to 100% B in 4 min, and maintained for 10 min more. The eluted peptides were introduced directly into a Q Exactive Plus Orbitrap instrument. Ionization was achieved by applying 1.9 kV to the source and setting the capillary temperature to 250 °C. The alternate current (radiofrequency) level of the S-lenses was set to 60 V. The complete MS1 scans (300 to 1500 *m*/*z*) were acquired in the profile mode with one micro-scan at 70,000 resolution and an automatic gain control (AGC) target value of 1 × 10^6^ with a maximum injection time of 100 ms. The 12 most intense precursor ions within both isolation window and offset of 2.0 and 0.5 *m*/*z*, respectively, were selected for higher-energy collision dissociation fragmentation with collision energy normalized to 30 units. The MS2 spectra (200 to 2000 *m*/*z*) were acquired in centroid mode with one micro-scan at 17,500 resolution and an AGC target value of 5 × 10^4^ with a maximum injection time of 50 ms. Dynamic exclusion was set to 30 s, whereas peaks with unassigned charges or those with z = 1 were rejected.

### 2.8. Protein Identification and Quantitation following Mass Spectrometry

Data were analyzed using the PatternLab for Proteomics V pipeline [33] available at http://www.patternlabforproteomics.org/ (accessed on 13 March 2023). An NCBI *H. capsulatum* database was downloaded on 2 December 2021 (95,405 entries). The “Generate Search Database” tool was used to remove (subset) sequences with 100% sequence identity (38,144 entries removed) and add common contaminants and decoy (reverse) sequences for all entries to a final database size of 114,768 entries. MS data from each run were searched against this database using the Comet search tool [34], integrated into PatternLab. The parameters were set as follows: precursor mass error tolerance of 35 ppm, trypsin as the enzyme, semi-tryptic cleavage, and a maximum number of cleavage loss of 2. Cysteine carbamidomethylation was set as a fixed modification, while deamidation of asparagine or glutamine, carbamidomethylation of aspartic acid, glutamic acid, histidine, and lysine, as well as methionine oxidation, were set as variable modifications. Settings for fragment bin tolerance, fragment bin offset, and theoretical fragment ions were 0.02, 0, and “use flanking peaks”, respectively. Spectral validation was performed using the SEPro algorithm [35]; the false discovery rate (FDR) was calculated from the number of decoy sequences identified so that a maximum limit of 1% FDR, at peptide and protein levels, was established, and only identifications with a mass error tolerance of 10^5^ ppm were accepted. Extracted ion chromatogram (XIC) was used for the quantitative comparisons between the two experimental groups in the TFold module [36] of PatternLab for Proteomics; normalization was done by total ion count. Only proteins that were detected in at least three (out of five) biological replicates, for each condition, and displayed at least 2 unique peptides, were submitted to relative quantitation with cutoff values for Benjamini–Hochberg q-value, F-stringency, and L-stringency of 0.05, 0.04, and 0.00, respectively. Proteins that satisfied the fold-change and statistical (*p*-value) criteria were further submitted to protein interaction and metabolic pathway analyses. The mass spectrometry proteomics data have been deposited to the ProteomeXchange Consortium (http://proteomecentral.proteomexchange.org, accessed on 13 March 2023) via the PRIDE partner repository [37] and received the dataset identifier PXD033762.

### 2.9. Bioinformatic Analyses

The Uniprot resource for protein sequence and annotation, available at https://www.uniprot.org/ (accessed on 13 March 2023), was used to describe the functions of proteins with quantitative differences in both conditions of cultivation of *H. capsulatum*. Enrichment analyses of gene ontology (GO) terms and Kyoto Encyclopedia of Genes and Genomes (KEGG) pathways were used to functionally categorize the regulated proteins. This analysis was performed on FungiDB [38], available at https://fungidb.org/fungidb/ (accessed on 13 March 2023), using a *p*-value cutoff of 0.05. GO enrichment analyses were limited to GO slim terms and used both curated and computed evidence. KEGG enrichment used EC Exact Match Only and included incomplete EC numbers. The removal of redundant GO terms was performed using the Revigo tool [39], available at http://revigo.irb.hr/ (accessed on 13 March 2023). 

### 2.10. Measurement of Mitochondrial Metabolic Activity 

The viability of *H. capsulatum* yeast cells was assessed in a Neubauer chamber by optical microscopy after trypan blue staining (Sigma Aldrich, St. Louis, MO, USA). Mitochondrial activity was determined with the CyQUANT XTT assay (Thermo Fisher, Bend, OR, USA). Preparation of inoculum (1 × 10^7^ viable cells/mL) was performed as described above (Ham’s F12 medium supplemented with cysteine, with and without the addition of mebendazole) maintained at 36 °C for 72 h in a rotatory incubator at 150 rpm. Cells were washed with sterile PBS three times. One hundred microliters of inoculum suspension prepared from each condition were added to a 96-well plate (1 × 10^6^ viable cells/well). As controls, only culture medium and culture medium combined with mebendazole at the same concentration used in the other experiments were used. All conditions were evaluated in biological triplicates. A mixture of XTT reagent and electron coupling reagent was prepared at a ratio of 6 to 1. Seventy microliters of the mixture were added to each well. Then, the plate was incubated at 37 °C for 4 h in the dark in a 5% CO_2_ incubator. The optical densities (OD) were determined, in two technical replicates, using the SpectraMax^®^ Plus 384 Microplate Reader (Molecular Devices, Ismaning, Germany) at 450 nm and 660 nm. Mitochondrial activity was expressed by the decrease in OD (450–660 nm), discounting the average of the ODs of the controls. 

### 2.11. JC-1 Assay

The mitochondrial transmembrane electric potential (ΔΨm) was investigated using the JC-1 fluorochrome, which is a lipophilic cationic mitochondrial vital dye that exhibits accumulation in mitochondria in response to ΔΨm. The fluorochrome exists as a monomer at low concentrations, where the emission is 530 nm (green fluorescence), but at higher concentrations, it forms J-aggregates after accumulation in the mitochondria, where the emission is 590 nm (red fluorescence). To this, *H. capsulatum* yeasts (10^6^ viable cells), treated or not with mebendazole for 72 h were harvested, washed in PBS, and incubated in a reaction medium containing 125 mM sucrose, 65 mM KCl, 10 mM HEPES/K^+^, pH 7.2, 2 mM Pi, 1 mM MgCl_2_, and 500 μM EGTA. To evaluate the ΔΨm, 10^6^ fungal cells were incubated with 10 μg/mL JC-1 solution for 30 min with readings made every minute using a microplate reader (SpectraMax spectrofluorometer, Molecular Devices). As positive control of the depolarization of the mitochondrial membrane, fungal cells were incubated with carbonyl cyanide 4-(trifluoromethoxy)phenylhydrazone (FCCP) at 1 μM, a mitochondrial protonophore. The relative ΔΨm value was obtained by calculating the ratio between the reading at 590 nm and the reading at 530 nm (590:530 ratio).

### 2.12. Size Measurements

*H. capsulatum* yeast cells were visualized with an AXIO Lab.A1 light microscope (ZEISS, Jena, Germany). The size of 100 cells was measured in ImageJ 1.40 g software (http://rsb.info.nih.gov/ij/, accessed on 13 March 2023; National Institutes of Health (NIH), Bethesda, MD, USA). The measurements were performed in three independent experiments.

### 2.13. Western Blot Analysis of Cytoskeleton Proteins

Proteins from *H. capsulatum* yeast-like cells, treated or not with mebendazole, were separated by 12% SDS-PAGE [32], and electrotransferred to 0.2 μm nitrocellulose membranes (Bio-Rad, Feldkirchen, Germany). The membrane strips were blocked at room temperature for 1 h in PBS containing 5% (*w*/*v*) non-fat skim milk and supplemented with 0.2% Tween 20 (pH 7.2) (T-PBS). The membranes were then washed three times with T-PBS (5 min per wash). Next, each membrane strip was incubated overnight at 4 °C with antibodies diluted in T-PBS: (a) monoclonal antibody (mAb) anti-beta-tubulin [1/1000 (*v*/*v*)] (protein molecular mass 50 kDa) [Sigma Aldrich, St. Louis, MO, USA] or (b) mAb anti-actin [1/1000 (*v*/*v*)] (protein molecular mass of 42 kDa) [Sigma Aldrich, St. Louis, MO, USA]. Three 5 min washes with T-PBS followed this step. The membrane strips were incubated as described above with horseradish-peroxidase conjugated anti-mouse IgG (Jackson ImmunoResearch, West Grove, PA, USA) diluted in T-PBS (1:3000) at 0.16 μg/mL. Following incubation, the membrane strips were washed and developed with SuperSignal West Dura Chemiluminescent substrate (Pierce, Rockford, IL, USA). Lastly, the X-ray films were exposed and developed according to the manufacturer’s instructions (Kodak, Rochester, NY, USA). The experiment was performed twice, on different days.

### 2.14. Statistical Analyzes

Data were analyzed using Prism 9 for Windows software (GraphPad Software LCC, San Diego, CA, USA). Initially, the normality of the data was verified using the Shapiro-Wilk test. Then, the parametric unpaired Student’s *t* test was used to compare results obtained with experiments that evaluated mebendazole-treated and -untreated conditions. For experiments with more than two groups to compare, two-way ANOVA was used. A significance level of 5% was employed in all analyses.

## 3. Results

### 3.1. Drugs with Anti-H. capsulatum Activity in the NIH Clinical Collection

**Figure 1:**
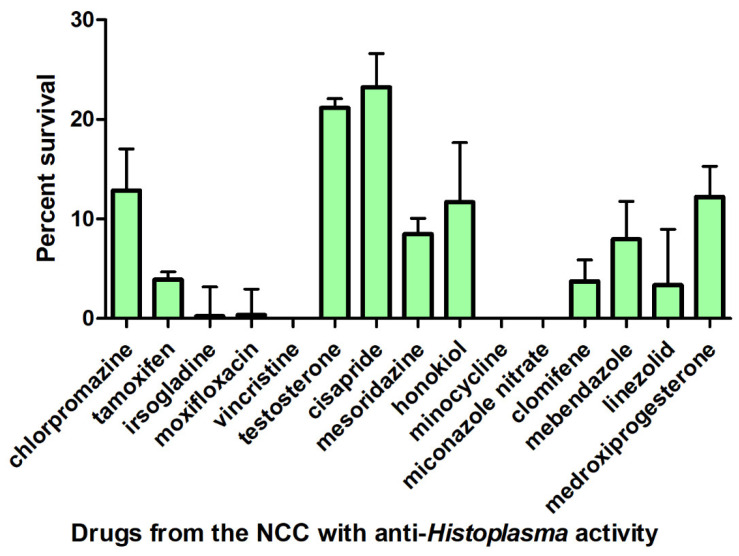
Antifungal activity of 15 drugs present in the NIH Clinical Collection (NCC) against the reference *H. capsulatum* G271B strain. Bars represent the mean and the standard deviation of the percent survivals determined in three independent experiments, as compared to the drug-free control wells. Only NCC drugs presenting less than 50% survival are presented.

From the 727 small molecules present in the NCC, 15 (2.1%), at 10 µM, consistently decreased survival by at least 50% of the *H. capsulatum* G217B strain after incubation at 37 °C for 72 h (Figure 1). Hit drugs were as follows: chlorpromazine, cisapride, clomifene, honokiol, irsogladine, linezolid, mebendazole, medroxyprogesterone, mesoridazine, miconazole, minocycline, moxifloxacin, tamoxifen, testosterone, and vincristine.

### 3.2. Mebendazole Antifungal Activity against H. capsulatum

During MIC determinations, we observed that chlorpromazine, irsogladine maleate, mesoridazine, and honokiol did not present satisfactory activity against the *H. capsulatum* strains included in this study (MIC ≥ 20 µM). We further investigated the drugs linezolid, mebendazole, and moxifloxacin, due to their antimicrobial activity and consequently better repurposing chances for histoplasmosis. MIC of these drugs against 11 *H. capsulatum* strains were consistent in all three experiments and are presented in Table 1. Only mebendazole consistently inhibited all fungal strains (geometric mean = 1.17 µM) and, therefore, it was selected for further studies.

Next, we evaluated whether mebendazole has fungicidal or fungistatic activity against *H. capsulatum* in three independent experiments, with similar results. Table 2 depicts the minimal fungicidal concentration of mebendazole against the 11 strains studied here. Mebendazole was fungistatic (MFC/MIC ratio > 2) for seven strains and fungicidal (MFC/MIC ratio ≤ 2) for four. 

### 3.3. Proteomics of Mebendazole-Treated H. capsulatum

**Figure 2:**
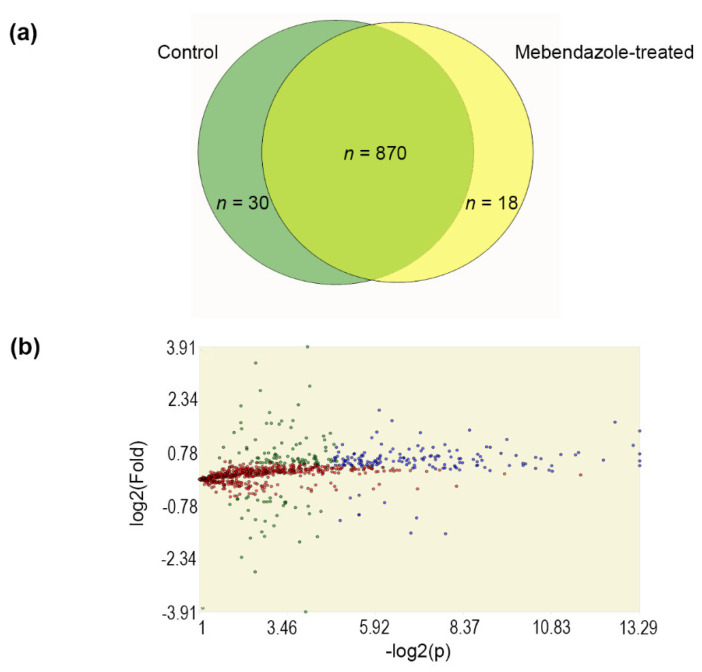
Proteomics of mebendazole-treated *H. capsulatum*: (**a**) Venn diagram showing the number of proteins present, in both technical replicates of at least three biological replicates, of control proteome, with DMSO (green) and mebendazole-treated proteome (yellow) that were detected in both technical replicates of at least three biological replicates; (**b**) Volcano plot of 870 proteins present in both proteomes. Blue dots indicate proteins that satisfied fold and statistical criteria (*n* = 159), green dots indicate proteins that satisfied the fold criteria but, most likely, this happened by chance (*n* = 121), and red dots indicate proteins whose identifications did not meet the fold and *p*-value criteria (*n* = 590). Green and red proteins were excluded from further analyses.

Proteomics identified 900 and 888 protein entries in untreated and mebendazole-treated (at ½ × MIC, for 72 h at 37 °C) cells of *H. capsulatum* G217B strain, respectively. These proteins were detected in both technical replicates of at least three biological replicates. Our proteomic approach detected 30 proteins exclusively in untreated fungal cells. Similarly, 18 proteins were only detected in the proteome of mebendazole-treated cells (Figure 2A). Among the proteins detected in both proteomes, 159 presented a statistically-supported (*p*- and *q*-values < 0.01) differential abundance between the two conditions tested (Figure 2B); 150 were more abundant in untreated cells and nine in mebendazole-treated cells (Table S1—“Blue” sheet). 

**Figure 3:**
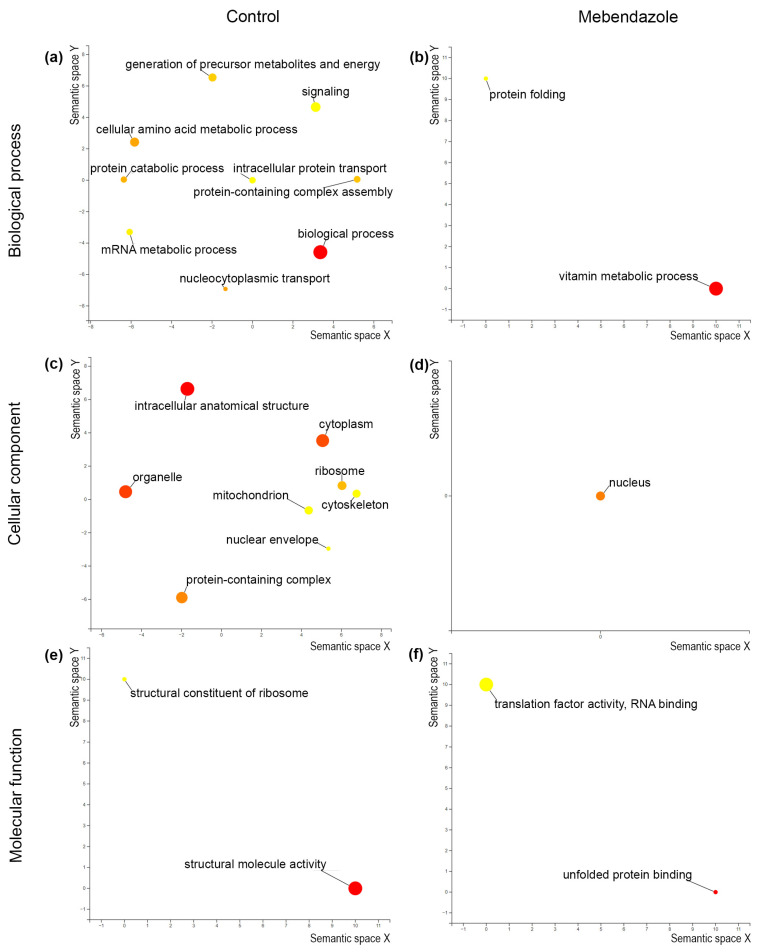
Enriched GO Slim terms in control and mebendazole-treated *H. capsulatum*: biological function (**a**,**b**); cellular component (**c**,**d**) and molecular function terms (**e**,**f**) are presented in control (**a**,**c**,**e**) and mebendazole-treated (**b**,**d**,**f**) proteomes. Revigo uses Multidimensional Scaling to reduce the dimensionality of a matrix of the GO terms’ pairwise semantic similarities. The resulting projection may be highly non-linear and the axes have no intrinsic meaning. The guiding principle is that semantically similar GO terms should remain close together in the plot.

The proteins with differential abundance and presenting exclusive detection considering both proteome conditions were clustered and analyzed using FungiDB. GO enrichment coupled with Revigo analyses revealed nine and two enriched biological processes, eight and one cellular components, and two and two enriched molecular function GO terms in the untreated and mebendazole-treated *H. capsulatum* extracts, respectively. Figure 3 depicts these GO terms, visualized through semantic similarity-based scatterplots. 

Table 3 shows the enriched metabolic pathways observed in proteomes from mebendazole-treated and untreated *H. capsulatum* yeast-like cells. Two pathways were enriched in the control (without mebendazole) condition, while three were enriched when the cells were treated with a subinhibitory dose of mebendazole.

### 3.4. Mebendazole Affects Mitochondrion Activity and H. capsulatum Cytoskeleton Proteins

**Figure 4:**
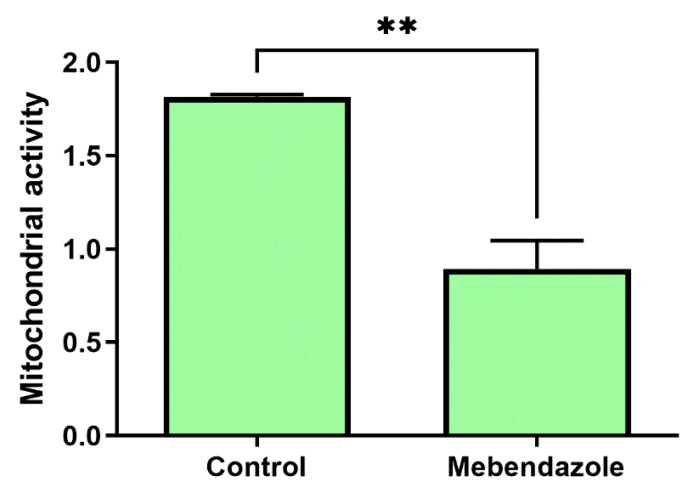
Mitochondrion metabolic activity of the *H. capsulatum* G271B strain after mebendazole treatment, as detected by the XTT assay. Mitochondrial activity was expressed by the difference in optical densities (OD) (450–660 nm), discounting the average of the OD of the blank (no fungal cells). Bars represent the mean and the standard deviation of three independent experiments read in duplicate. ** *p* < 0.01 (Student *t* test).

To confirm that mebendazole decreases mitochondrion activity, as suggested by the metabolic pathway enrichment analysis that indicated a citrate cycle enrichment in cells not treated with mebendazole, the XTT assay was performed in the same number of viable *H. capsulatum* cells, as demonstrated by trypan blue exclusion test of cell viability. Figure 4 shows the XTT assay in untreated and mebendazole-treated *H. capsulatum* yeast-like cells, showing that mitochondrion metabolic activity was lower in mebendazole-treated cells (*p* = 0.0037). 

**Figure 5:**
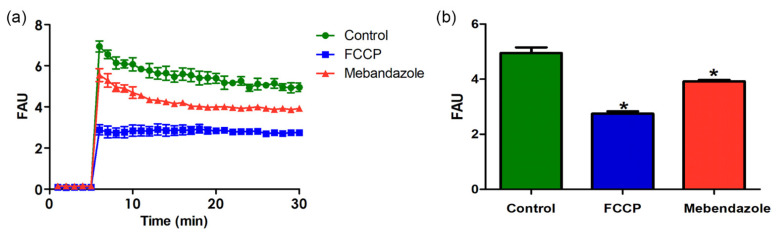
The ΔΨm analysis of control (with DMSO only) and *H. capsulatum* treated with mebendazole was performed using the JC-1 fluorochrome for 30 min. FCCP (1 μM) was used as a positive control of the depolarization of the mitochondrial membrane. (**a**) Comparisons of the ΔΨm values between the different systems in different time points and (**b**) after 30 min of reaction. The results are expressed as fluorescence arbitrary units (FAU). Values represent the mean ± standard deviation of three independent experiments, performed in triplicate. The asterisk represents significant statistical differences (* *p* < 0.05) (Two-way ANOVA).

The maintenance of mitochondrial membrane potential is essential for mitochondrial function. Mebendazole-treated *H. capsulatum* yeast-like cells had a significant reduction (*p* < 0.05) in the ΔΨm compared to untreated cells after 30 min of reaction (Figure 5). In addition, incubation with the classical inhibitor of the mitochondrial function (FCCP) completely collapsed the ΔΨm of the *H. capsulatum*. 

**Figure 6:**
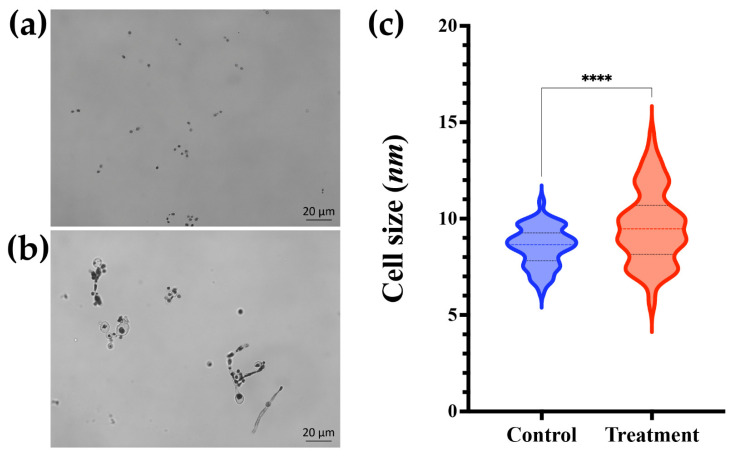
Influence of mebendazole in the *Histoplasma capsulatum* morphology. (**a**) *H. capsulatum* control; (**b**) *H. capsulatum* treated with mebendazole at optical microscopy (Bars: 20 µm) and (**c**) Cellular diameter size *of H. capsulatum* yeast cells, treated with mebendazole and control (DMSO only). Microscopy images are representative of three independent experiments. The color lines inside the violins represent the median diameter size and the black lines the interquartile range of 100 measurements performed in triplicate. (**** *p* < 0.0001, Student’s *t* test).

**Figure 7:**
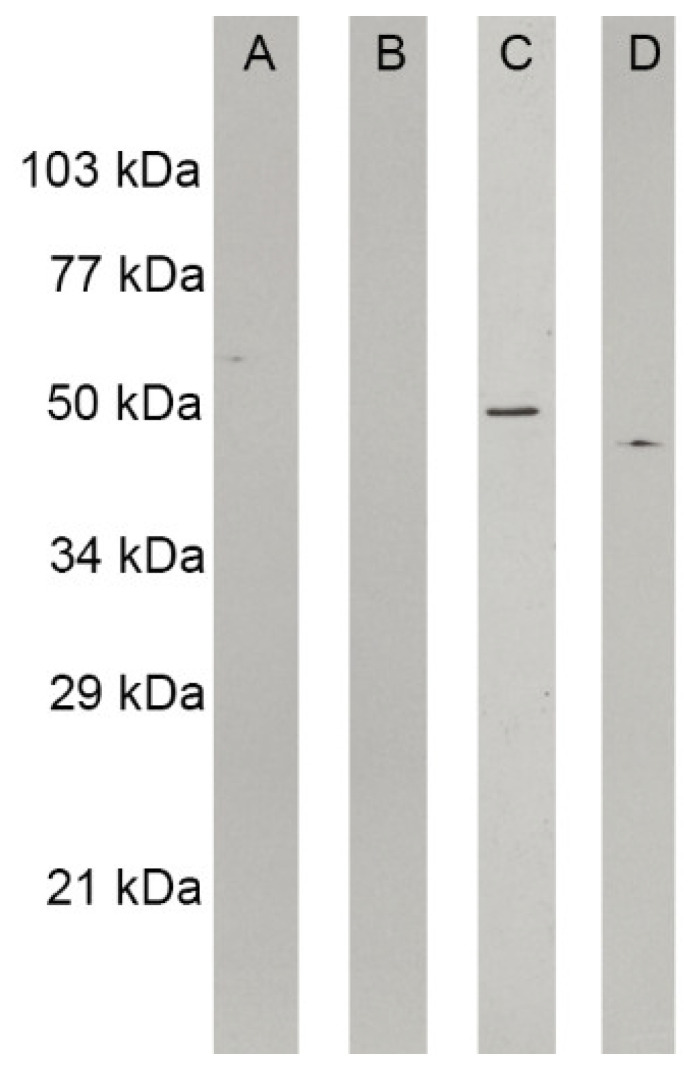
Western blot analyses of structural proteins on *H. capsulatum* protein extracts. Strips were revealed with commercial anti-actin or anti-tubulin monoclonal antibodies: (**A**) mebendazole-treated extract revealed with anti-tubulin antibody; (**B**) mebendazole-treated extract revealed with anti-actin antibody; (**C**) mebendazole-untreated extract revealed with anti-tubulin antibody, with a positive 50 kDa band; (**D**) mebendazole-untreated extract revealed with anti-actin antibody, with a weekly positive 42 kDa band.

*Histoplasma capsulatum* yeast cells treated with mebendazole showed a larger size when compared to the untreated ones (*p* < 0.05) (Figure 6). In addition, to evaluate whether mebendazole decreases cytoskeleton proteins, as suggested by the GO enrichment analysis that indicated an enrichment of cytoskeleton in the cellular component ontology and an enrichment of structural molecule activity in the molecular function ontology, we performed a western blot analysis using both untreated and mebendazole-treated protein extracts and commercial mAbs against actin and tubulin. The proteome data indicated a 1.35 fold-change of actin and an exclusivity of α-tubulin in the mebendazole-free extract (Table S1). As demonstrated by the western blot, both actin and tubulin were not detected in the mebendazole-treated extract. In addition, a positive 50 kDa band appeared in the mebendazole untreated proteome, which has a molecular weight compatible with the tubulin beta chain (QSS49519) protein present in this extract. Moreover, a weekly positive 42 kDa band also appeared when treated with the other antibody, and this protein has a molecular weight compatible with the actin (KAG5301831) protein detected in the untreated proteome (Figure 7). The absence of an actin band in the mebendazole-treated extract may be explained by a concentration below the detection limit of the method. 

## 4. Discussion

The development of new medicinal drugs is long, complex, and costly, requiring numerous studies during the course of all phases of clinical research [40]. A more accessible alternative is drug repurposing, a strategy to identify new uses for existing and marketed drugs for treatments different from those initially described [41]. Some studies point to promising drug repurposing results for *Histoplasma* [19,20], *Cryptococcus* [26,42], and *Paracoccidioides* infections [43]. In fact, due to the paucity of currently approved antifungal drugs, more repurposing studies for these life-threatening pathogens are necessary.

One of the experimental approaches for drug repurposing studies is phenotypical screening, which identifies substances with disease-relevant effects in in vitro models without prior knowledge of the affected target [41]. In this study, we evaluated the NCC against *H. capsulatum*, a human pathogenic fungus with a very low number of approved medicines to combat its disease [4,5]. The NCC was previously used for phenotypical screening of antifungal activity against *C. neoformans*, where mebendazole was also the drug with the most promising repurposing results [26]. In addition to mebendazole, our study also revealed 14 other drugs that consistently inhibited *H. capsulatum* growth. One of them, miconazole, is a topical imidazole [44], without relevance in the context of severe disseminated disease. Four are sexual hormones or act on sexual hormone receptors (tamoxifen, testosterone, clomifene, and medroxyprogesterone), which makes their general use on both sexes difficult. Tamoxifen also has known antifungal activity against *C. neoformans* and *Candida* species [45]. Cisapride was removed from the market, due to its severe cardiac adverse effects [46]. Vincristine sulfate, a vinca alkaloid used to treat acute leukemia, malignant lymphoma, Hodgkin’s disease, acute erythraemia, and acute panmyelosis, presents serious life-threatening adverse interactions with itraconazole [47] and neurotoxic side-effects [48], discouraging its use, solely or in combination, to treat histoplasmosis. Two antipsychotic drugs (chlorpromazine and mesoridazine) as well as honokiol and irsogladine maleate were not active against the strains herein studied during MIC determinations. Therefore, we excluded these drugs from further analyses.

The remaining active drugs observed during the NCC screening are used to treat non-fungal infectious diseases: three antibiotics (moxifloxacin, minocycline, and linezolid) and the benzimidazolic anthelmintic mebendazole. Their use to treat other infectious diseases is interesting in the context of histoplasmosis, since their safety, tolerability, and dosages are well known. We were not able to test the MIC of minocycline, which is a limitation of the current study. Minocycline is synergistic with several azoles against a multitude of *Candida* species [49]. Its anti-*H. capsulatum* activity remains to be elucidated. Unfortunately, moxifloxacin and linezolid were not active against all tested strains, which limits their potential to be repurposed to treat histoplasmosis. Moxifloxacin reduces in vitro growth and virulence of *Candida albicans* [50] and linezolid is effective against *Pythium insidiosum* [51]. However, our results encourage their use when co-infections caused by bacteria sensitive to these drugs occur in patients with histoplasmosis.

Mebendazole was first approved to treat intestinal helminthiasis, and high doses are recommended by the World Health Organization to treat echinococcosis. Furthermore, this benzimidazole has been extensively studied for drug repurposing in oncology [52]. Mebendazole has antifungal activity against *C. neoformans*, being fungicidal against the reference H99 strain [26]. Here, mebendazole was fungistatic against the reference G217B *H. capsulatum* strain and the majority of the other strains. Another limitation of the present study is the lack of *H. capsulatum* strains from outside Brazil. Since *H. capsulatum* harbors several phylogenetic species highly associated with the geographic region of isolation [24], the mebendazole anti-*H. capsulatum* activity may be different, depending on the genetic background of the fungus.

An interesting fact regarding mebendazole repurposing for histoplasmosis is its anti-*Histoplasma* and anti-*Cryptococcus* activity. There are reports of co-infections between these two fungi in PLWHA [53,54,55,56] and a new medicine targeting both agents is attractive. Another important point regarding mebendazole repurposing for histoplasmosis is that this benzimidazole has good blood–brain barrier penetration in an experimental model of infection, with concentrations up to 7.1 µM [57], which is higher than all mebendazole MIC determined in this study. This would be important for *H. capsulatum* meningitis that, although rare, needs prompt and effective therapy for efficient management [58].In addition, mebendazole induced an apparent increase in the cell size of *Histoplasma capsulatum* yeast cells when compared to the control, a factor that may contribute to the spread of this pathogen and may be an escape mechanism from phagocytosis. Cellular ultrastructure studies should be used to visualize morphological alterations and virulence studies in strains related to this drug. A proteomic approach was used in this study to understand metabolic changes driven by mebendazole on *H. capsulatum* yeast-like cells, which interestingly appears to be similar to the mebendazole mechanism of action in other cell types. Mebendazole is known to bind the β-subunit of tubulin, hampering dimerization with α-tubulin [52]. Both actin and tubulin were less abundant in mebendazole-treated *H. capsulatum* cells, as demonstrated by proteomics and the western blot using monoclonal antibodies for these proteins. In addition, mebendazole disrupts certain enzymes of the carbohydrate metabolism in some Cestoda parasites [59]. Proteins from the citrate cycle were significantly less abundant in the mebendazole-treated *H. capsulatum* extract, as demonstrated by proteomics and by a lower mitochondrial activity of viable mebendazole-treated *H. capsulatum*. Previously, it was reported that mebendazole promoted the mitochondrial cytochrome c release in lung cancer cells, affecting mitochondrial function [60,61]. The treatment of chronic myeloid cells (K562 and FEPS) [62] and colon cancer cell line HT29 [63] with mebendazole was able to induce loss of mitochondrial viability. Similarly, in the present work, we verified through the JC-1 technique, which is a tool to monitor mitochondrial health, that the mebendazole treatment significantly reduced the mitochondrial membrane potential of *H. capsulatum* cells. Similarly, XTT assay revealed a significant decrease in the enzymatic activity of mitochondrial dehydrogenases. In consonance, both previous experiments corroborated the mitochondrial dysfunction in *H. capsulatum* cells induced by the mebendazole treatment. The proteomic results also pointed to a lower abundance of ribosome-related proteins after mebendazole treatment. In fact, a significant reduction in ribosome numbers occurs in the microsporidian fungus *Glugea anomala* after mebendazole treatment [64]. This ribosome-related mechanism of action of mebendazole in *H. capsulatum* needs further studies.

Another relevant limitation of this study is the lack of in vivo studies to ascertain whether mebendazole is effective during *H. capsulatum* infection.

## 5. Conclusions

The results herein suggest the use of mebendazole as a promising candidate drug to be repurposed for histoplasmosis treatment. Mebendazole acts on *H. capsulatum* by inhibiting oxidative metabolism, disrupting the cytoskeleton, and probably affecting fungal ribosomes. Future clinical trials are needed to validate mebendazole as a new drug to treat histoplasmosis.

## Figures and Tables

**Table 1:** Minimal inhibitory concentrations (consistent results from three independent experiments) of three antimicrobial drugs with anti-*H. capsulatum* activity.

*H. capsulatum* Strain	Linezolid	Mebendazole	Moxifloxacin
G217B	10 µM	2.5 µM	10 µM
IGS 4/5	>20 µM	1.25 µM	>20 µM
2690603	>20 µM	0.08 µM	>20 µM
CE 17/13	>20 µM	0.08 µM	20 µM
GO 11/15	20 µM	2.5 µM	20 µM
M477/08	10 µM	2.5 µM	>20 µM
CAO4	2.5 µM	0.6 µM	>20 µM
IPEC 23/11	0.3 µM	2.5 µM	>20 µM
CE12/14	>20 µM	5.0 µM	>20 µM
39942	>20 µM	1.25 µM	> 20 µM
20258-1	>20 µM	5.0 µM	>20 µM

**Table 2:** Fungicidal activity, determined in three independent experiments, of mebendazole against 11 *H. capsulatum* strains.

*H. capsulatum* Strain	MFC ^1^	MFC/MIC ^2^ Ratio
G217B	>20 µM	>8
IGS 4/5	>20 µM	>16
2690603	1.25 µM	16
CE 17/13	2.5 µM	32
GO 11/15	2.5 µM	1
M477/08	2.5 µM	1
CAO4	2.5 µM	4
IPEC 23/11	2.5 µM	1
CE12/14	>20 µM	>4
39942	1.25 µM	1
20258-1	>20 µM	>4

^1^ MFC: Minimal fungicidal concentration. ^2^ MIC: Minimal inhibitory concentration.

**Table 3:** Metabolic pathway enrichment analysis of proteomes derived from untreated and mebendazole-treated *H. capsulatum* G217B yeast-like cells.

Proteome Condition	KEGG Metabolic Pathway ^1^	Odds Ratio ^2^	*p* Value ^2^
Without mebendazole	Aminoacyl-tRNA biosynthesis	3.06	0.0215
	Without mebendazole>Citrate cycle	Without mebendazole>3.21	Without mebendazole>0.0472
Treated with mebendazole	Pyrimidine metabolism	9.49	0.00814
	Nitrogen metabolism	12.47	0.0167
	Sulfur metabolism	15.17	0.0117

^1^ KEGG: Kyoto encyclopedia of genes and genomes. ^2^ Odds ratio and *p* value from the Fisher’s exact test.

## Data Availability

Publicly available datasets were analyzed in this study. This data can be found here: http://proteomecentral.proteomexchange.org (accessed on 13 March 2023), under the identifier PXD033762.

## Supplementary Materials

The following supporting information can be downloaded at: https://www.mdpi.com/article/10.3390/jof9030385/s1, Table S1: Proteins with differential abundance in *Histoplasma capsulatum* G217B proteomes and their classification after Benjamini–Hochberg q-value, F-stringency, and L-stringency analyses.
